# Multi-Source and Multi-Representation Adaptation for Cross-Domain Electroencephalography Emotion Recognition

**DOI:** 10.3389/fpsyg.2021.809459

**Published:** 2022-01-13

**Authors:** Jiangsheng Cao, Xueqin He, Chenhui Yang, Sifang Chen, Zhangyu Li, Zhanxiang Wang

**Affiliations:** ^1^School of Informatics, Xiamen University, Xiamen, China; ^2^Department of Neurosurgery, The First Affiliated Hospital of Xiamen University, Xiamen, China; ^3^Department of Neurosurgery, Xiamen Key Laboratory of Brain Center, The First Affiliated Hospital of Xiamen University, Xiamen, China; ^4^Department of Neuroscience, Institute of Neurosurgery, School of Medicine, Xiamen University, Xiamen, China

**Keywords:** EEG, emotion recognition, domain adaption, deep learning, affective computing, SEED

## Abstract

Due to the non-invasiveness and high precision of electroencephalography (EEG), the combination of EEG and artificial intelligence (AI) is often used for emotion recognition. However, the internal differences in EEG data have become an obstacle to classification accuracy. To solve this problem, considering labeled data from similar nature but different domains, domain adaptation usually provides an attractive option. Most of the existing researches aggregate the EEG data from different subjects and sessions as a source domain, which ignores the assumption that the source has a certain marginal distribution. Moreover, existing methods often only align the representation distributions extracted from a single structure, and may only contain partial information. Therefore, we propose the multi-source and multi-representation adaptation (MSMRA) for cross-domain EEG emotion recognition, which divides the EEG data from different subjects and sessions into multiple domains and aligns the distribution of multiple representations extracted from a hybrid structure. Two datasets, i.e., SEED and SEED IV, are used to validate the proposed method in cross-session and cross-subject transfer scenarios, experimental results demonstrate the superior performance of our model to state-of-the-art models in most settings.

## Introduction

Emotion is a physiological state of humans, which appears when people are stimulated by external or their own factors. Emotion is the basis of human daily life and work and plays an important role in human psychological development, interpersonal communication, rational decision-making, and cognition. Correct recognition of emotions is of great significance in the fields of education, medical treatment, psychology, cognitive science, and artificial intelligence. For example, in the medical field, emotion recognition can help doctors diagnose and treat patients with expression disorders; in the education field, through emotion recognition, teachers can quickly find and encourage students with poor emotions to create a more positive learning environment.

Data sources used for emotion recognition can be roughly divided into two categories: non-physiological signals and physiological signals. Many scholars have carried out researches on emotion recognition using non-physiological signals such as gestures, facial expressions, eye expressions, and voice. Said and Barr (2021) proposed a face-sensitive convolutional neural network (FS-CNN) to detect human emotion. Trigeorgis et al. (2016) utilized a convolutional recurrent model based on the raw signal to achieve end-to-end spontaneous emotion prediction. In addition, some scholars (Camurri et al., 2003; Durupinar et al., 2016; Senecal et al., 2016) try to identify emotions from dance movements. These methods are limited to specific dance moves and lack practical significance. Due to the easy camouflage of non-physiological signals, those non-physiological signal-based emotion recognition methods are unstable, and the recognition effect is easily affected by subjective factors. Compared with non-physiological signals, physiological signals [such as blood pressure (BVP), electroencephalogram (EEG), electrooculogram (EOG), electrocardiography (ECG), electromyogram (EMG), etc.] are spontaneously generated by the human body and can truly reflect the emotional state of humans, which has high reliability. Therefore, scholars have shifted the focus of research to emotion recognition using physiological signals. Among physiological signals, EEG, which has the characteristics of non-subjectivity, real-time difference, and rich information, has been widely used in the field of emotion recognition. Studies have shown that EEG played an important role in the research of human emotion and that regional brain activity was closely related to some emotional states (Niemic, 2004).

With the continuous development of artificial intelligence technology, artificial intelligence has also shown a broader prospect in the field of emotion recognition based on EEG. Employing emerging intelligent technologies like machine learning and computer vision to realize EEG-based quantitative analysis and recognition of emotions has become a research hotspot. Existing EEG-based emotion recognition methods can be roughly divided into two categories: traditional machine learning-based methods and deep learning-based methods. Among the methods based on traditional machine learning, Support Vector Machine (SVM) (Anh et al., 2012; Moshfeghi et al., 2013; Zulkifli et al., 2015), Bayesian network (Yoon and Chung, 2013), K-means algorithm (Mohanty and Swain, 2011), decision tree algorithm, K-Nearest Neighbor (KNN) (Heraz and Frasson, 2007) and other classifiers are exploited for emotion classification and recognition. However, traditional machine learning highly relies on manual feature extraction, which is time-consuming and labor-intensive. Because of the advantages of deep learning technology in automatic feature extraction, many EEG-based emotion recognition methods based on deep neural networks are proposed, such as Yang et al. (2018) using a hybrid neural network which combines ‘Convolutional Neural Network (CNN)’ and ‘Recurrent Neural Network (RNN)’ to effectively learn the compositional spatial-temporal representation of raw EEG streams, and Song et al. (2018) utilized the dynamical graph convolutional neural network (DGCNN) to model multichannel EEG features and then classify the EEG emotion.

Since EGG signals are subject-specific, there are large variations between individuals (Jayaram et al., 2015). To solve the problem, Hwang et al. (2020) proposed an adversarial learning method for subject-independent EEG-based classification methods and achieved good performance on the SEED data set. Li et al. (2019a) proposed a multisource transfer learning method for cross-subject EEG emotion recognition, which can generalize existing models to a new person. But this method did not consider the invariant features of the domain, which would lead to the loss of part of the information. Therefore, many scholars introduce domain adaptation (DA) into deep learning models to obtain domain-invariant representations and achieve remarkable results. Zhu D. et al. (2019) provided a two-stage alignment framework for deep learning methods based on Multi-source Unsupervised Domain Adaptation (MUDA), which not only aligns the domain-specific distributions of each pair of source and target domains in multiple feature spaces to learn multiple domain-invariant representations but also align the domain-specific classifiers’ output for target samples. This type of approach (Long et al., 2016) mainly matches the embedding of distributions by introducing an adaptive layer (Zhu J. et al., 2019). Another mainstream method based on deep learning is to directly utilize adversarial methods or generative adversarial methods, such as Wasserstein generative adversarial network domain adaptation (WGANDA) (Luo et al., 2018), few-label adversarial domain adaption (FLADA) (Wang et al., 2021), joint distribution adaptation method (Li et al., 2019b), and so on. However, most of these methods are single-representation adaptation, which leads to the alignment of the source and target domains to concentrate on partial information (Zhu J. et al., 2019). Besides, due to the non-stationary between individual sessions (Sanei and Chambers, 2007), EEG-based emotion recognition still has the problem of cross-domain. Most of the existing studies aggregate the EEG data from different subjects and sessions into one source domain, ignoring the difference in the edge distribution of different EEG domains. Thus, how to achieve cross-subject and cross-session EEG-based emotion recognition is still a huge challenge.

To address these problems, we propose a cross-domain EEG emotion recognition method based on multi-source and multi-representation adaptation (MSMRA). In the data processing stage, the EEG signal is divided into multiple source domains to avoid destroying the edge distribution of the source domain data. Since the multi-source will complicate the network structure, we propose a two-partition method to aggregate two EEG data into one source domain. On this basis, the low-level domain-invariant features of different EEG data are extracted by the common feature extraction module. Then for multiple source domains, the multi-domain specific feature extractor (MDSFE) module enhances the representation capability of the source by aligning multiple representation distributions excavated from a hybrid structure. The multiple distributions are converted into a vector and fed into the classifier for final sentiment inference. Our extensive experiments on two public datasets show that MSMRA can achieve remarkable performance compared with state-of-the-art competitors.

The contributions of this paper are summarized as follows.

(1) We propose the MSMRA network to aggregate the EEG data of different subjects and sessions into multiple source domains, thereby improving the accuracy of emotion classification based on EEG in cross-domain scenarios.
(2) A two-partition method is proposed to divide EEG data into multiple source domains, which not only significantly reduces the redundancy of the network structure but also expands the data of a single source to improve the emotion classification accuracy.
(3) To obtain more high-level features of EEG signals, we constructed an MRA module to align the distribution of multiple different representations.
(4) We conducted extensive experiments on two public datasets to validate the performance of MSMRA compared with state-of-the-art methods.

## Materials

### BCI Process Flow for Emotion Recognition

**FIGURE 1:**
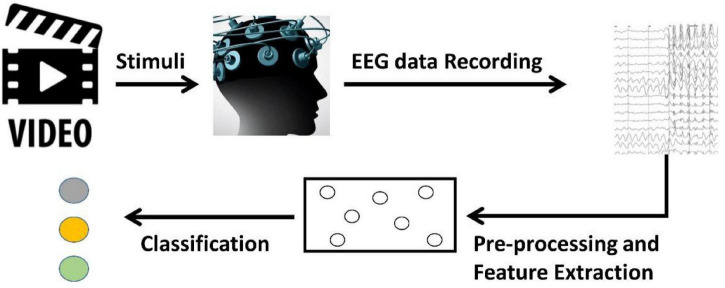
Process flow for brain-computer interfaces.

As observed in Figure 1, the BCI process flow for emotion recognition usually consists of four-steps (Vasiljevic and Miranda, 2020; Gu et al., 2021). The subject is first stimulated with some stimuli, which are often movie clips containing various emotions, then the electrodes placed in the subject’s skull are used to record the EEG signal. After data processing, the EEG data is finally fed into classification models.

### Datasets

To evaluate the proposed methods, we conduct experiments on two public datasets, namely SEED and SEED-IV (Duan et al., 2013; Zheng and Lu, 2015; Zheng et al., 2019). In the SEED dataset, a total of 15 subjects (7 males and 8 females) participated in the experiment. Their EEG data was collected *via* 62 EEG electrodes when they were watching 15 Chinese movie clips with negative, positive, and neutral emotions. To avoid subject fatigue, the entire experiment will not last for a long time, and the duration of each segment is about 4 min. Since the video can be understood without explanation and the video only triggers a single target emotion, all EEG signals will be classified into one of three emotional states (positive, neutral, and negative). The data collection lasts for 3 different periods at approximately 1-week intervals, corresponding to three sessions, so each subject has a total of 45 EEG data trials. In addition, to ensure that the collected EEG data and the movie clips presented to the subjects have the same emotional state, an additional subjective self-assessment will be performed on each subject after the subjects watch the movie. The relevant information about the SEED dataset is shown in Table 1.

**TABLE 1:** Information about SEED dataset.

Attributes	Details Information
Source	BCMI laboratory
Sessions	Three
Subjects	Fifteen
Trials	Fifteen
Emotions	Positive Neutral Negative
Channels of recorded	62 EEG channel

Similar to SEED, SEED-IV contains a total of 15 subjects (7 males and 8 females) of 62-channel EEG data. These data are the four types of emotions collected by the subjects when they watch emotion-induced movie clips, namely, neutral, sad, fear, and happy. Each movie clip lasts about 2 min. A total of three sessions are collected. Each session includes 24 trials/movies for each subject. Compared to SEED that only uses EEG signals, the database also includes eye movement features recorded by SMI eye-tracking glasses. The relevant information about the SEED-IV dataset is shown in Table 2.

**TABLE 2:** Information about SEED-IV dataset.

Attributes	Details Information
Source	BCMI laboratory
Sessions	Three
Subjects	Fifteen
Trials	Twenty four
Emotions	Neutral Sad Fear Happy
Channels of recorded	62 EEG channel

### Experimental Scenarios

Due to the non-stationarity of the EEG signals between sessions and subjects (Sanei, 2013), the EEG signals of different subjects in the same session and the EEG signals of the same subject in different sessions will have a certain degree of difference. Therefore, in the field of emotion recognition of EEG signals, domain adaptation can be divided into several situations:

#### Cross-Subject Transfer

In the same session, take the EEG data of the new last subject as the target domain, and the EEG data of all the remaining subjects as the source domain. In this article, since the SEED and SEED-IV datasets have a total of 3 sessions and 15 subjects, we will group the EEG data of the subjects in pairs to form a source field, there are 7 in total, and the final subject’s EEG data is used as the target domain.

#### Cross-Session Transfer

In different session experiments of the same subject, we combine the EEG data of the previous two sessions into a source domain, and the EEG data of the last session is regarded as the target domain (see section “Methods” for details).

### Electroencephalography Data Pre-processing

**FIGURE 2:**
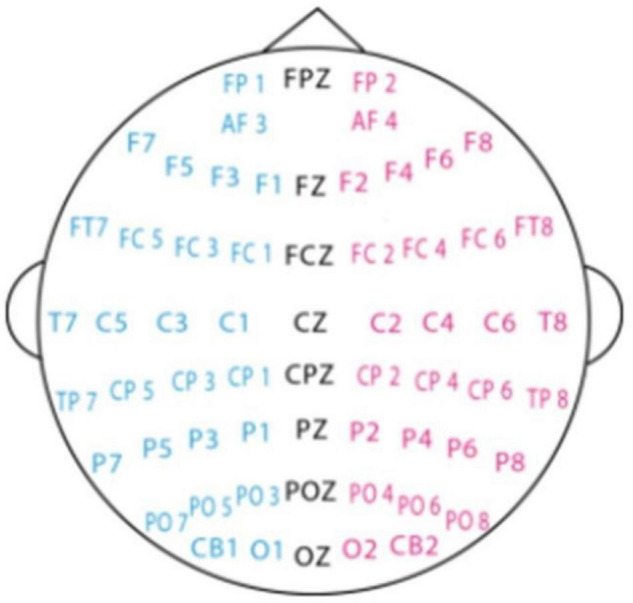
EEG electrode placement.

The collected data is first down-sampled to a sampling rate of 200 Hz, and then a band-pass frequency filter of 0–75 Hz is applied to filter noise and remove artifacts. After the EEG sequence is converted to the frequency domain, the differential entropy features of each frequency band (Delta, Theta, Alpha, Beta, and Gamma) and each channel (62 channels) are extracted (62 channel positions are shown in Figure 2), where the frequency of the Delta band is 1∼4 Hz, and the frequency of the Theta band is 4–8 Hz, the frequency of the Alpha band is 8–14 Hz, the frequency of the Beta band is 14–31 Hz, and the frequency of the Gamma band is 31–50 Hz.

The differential entropy feature is defined as Equation 1:


(1) $$\text{DE}=-\int_{-\infty }^{\infty }P\left(x\right)\ln\text{[}P\left(x\right)]dx$$

where DE represents the differential entropy features, *x* denotes a random variable, P(*x*) indicates the probability density of *x*.

We assume that the EEG signal obeys a normal distribution *x*N(,^2^), then the calculation of the DE feature can be simplified to Equation 2:


(2) $$\text{DE}=-\int_{-\infty }^{\infty }\frac{1}{\sqrt{2\,\pi \,\sigma }}\,\text{exp}\,\frac{{\left(x-\mu \right)}^{2}}{2\,\sigma^{2}}\,\ln\left(\frac{1}{\sqrt{2\,\pi \,\sigma }}\,\text{exp}\,\frac{{\left(x-\mu \right)}^{2}}{2\,\sigma^{2}}\right)\,dx$$

In this work, in both the Cross-Subject Transfer and Cross-Subject Transfer scenarios, we use DE features extracted from EEG raw data as input, and one data from one subject in one session for both databases is in the form of trials (SEED 15, SEED-IV 24) × channel (62) × band (5) × samples, we then merge the channel and frequency band data into trials (SEED 15, SEED-IV 24) × 310 × samples. For the SEED dataset, 15 movie clips contain a total of 3,394 samples corresponding to each session, and for the SEED-IV dataset, 24 movie clips contain a total of 851/832/822 samples corresponding to three sessions.

## Methods

**FIGURE 3:**
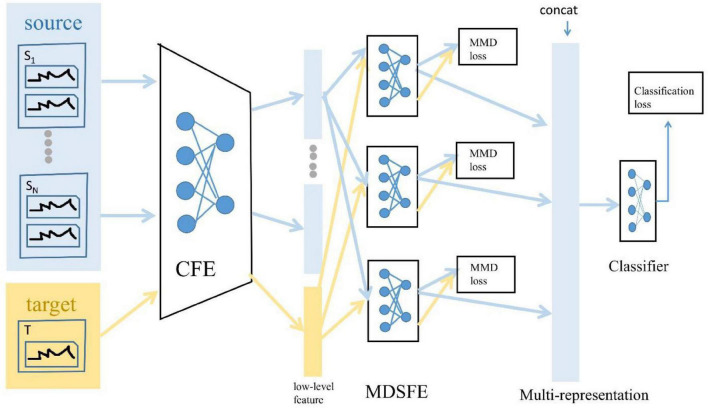
Architecture of proposed Multi-Source and Multi-Representation Adaptation (MSMRA) method.

There are two significant concepts in the domain adaptation: the source domain represents a domain different from the test sample but has rich supervision information, and the target domain represents the domain where the test sample is located, without or only have a few labels (Pan and Qiang, 2010). The source domain and the target domain often belong to the same task, but the distribution is different. In this article, we regard the newly collected EEG data as the target domain, and all the remaining previously collected EEG data as the source domain. Given the data of the source domain and target domain, we are not simply aggregating all EEG data into a source domain, which ignores the internal differences of EEG data of different subjects and different sessions (Zhu D. et al., 2019), in contrast, we combine two groups of EEG data into multiple source domains, which not only considers the internal differences of EEG data but also expands the number of samples of source domain data, which can prove to be effective in improving the accuracy of sentiment classification based on EEG. Moreover, we believe that just projecting the source domain and target domain into a common feature subspace cannot cover all information (Zhu J. et al., 2019), so we extract multiple representations from low-pixel images to obtain more information. The architecture is shown in Figure 3.

As shown in Figure 3, suppose we have N source domains, which can be defined as:


(3) $$\text{S}=\left\{{\text{S}}_{1},{\text{S}}_{2},\ldots ,{\text{S}}_{\text{N}}\right\}$$

Where S denotes the collection of source domains, S_N_ means Nth source domain. Each source domain consists of two different EEG data, which is expressed as follows:


(4) $$S_{i}={\left\{E_{2\,i-1}^{S},E_{2\,i}^{S}\right\}}_{i=1}^{N}$$

The target domain can be expressed as T = {*E^T^*}. The data of the source domain and the target domain are fed into a common feature extractor module c(⋅) to extract low-level feature of both ${\left\{F_{i}^{S}\right\}}_{i\,1}^{N}\,?\,F_{i}^{T}:$


(5) $$F_{i}^{S}=c\,\left(S_{i}\right)$$

(6) $$F_{i}^{T}=c\,\left(T\right)$$

Then the low-level features of the source domain and the target domain ${\left\{F_{i}^{S}\right\}}_{i=1}^{N}$ and $F_{i}^{T}$ are fed into the multi-domain specific feature extractor module $\{{\text{h}}_{\text{j}}^{\text{i}}\left(\cdot \right){\text{\}}}_{j=1}^{r}$ to obtain the multi-expression, respectively, ${\left\{R_{i}^{S}\right\}}_{i=1}^{N}$ and $R_{i}^{T}$.


(7) $$R_{i\,j}^{S}={\left\{{\text{h}}_{\text{j}}^{\text{i}}\,\left(F_{i}^{S}\right)\right\}}_{j=1}^{r}$$

(8) $$R_{i\,j}^{T}={\left\{{\text{h}}_{\text{j}}^{\text{i}}\,\left(F_{i}^{T}\right)\right\}}_{j=1}^{r}$$

The multi-domain-specific feature extractor has r different sub-domain-specific feature extractors, and each sub-domain-specific feature extractor can obtain various high-level feature expressions. Compared with a simple single representation, multiple representations can cover more information to better judge emotion classification. After obtaining the multiple representations of the source domain and the target domain ${\left\{R_{i}^{S}\right\}}_{i\,1}^{N}$ and $R_{i}^{T}$, the maximum mean discrepancy (MMD) loss of the different expressions of the source domain and the corresponding target domain expression can be calculated. The MMD loss is a measure of the distance between the current source domain and the target domain (Borgwardt et al., 2006), and the final MMD loss is the sum of the MMD loss of each source domain expression and the corresponding target domain expression. The MMD loss can be formulated as Equation 9, and the final MMD loss can be defined as Equation 10.


(9) $$M\,M\,D_{j}\,\left(R_{i\,j}^{S},R_{i\,j}^{T}\right)={||\frac{1}{n}\,\sum_{x=1}^{n}\phi \,\left(r_{x}^{S}\right)-\frac{1}{m}\,\sum_{y=1}^{m}\phi \,\left(r_{y}^{T}\right)||}_{H}^{2}$$

(10) $$M\,M\,D_{f\,i\,a\,n\,l}^{i}=\sum_{j=1}^{r}M\,M\,D_{j}\,\left(R_{i\,j}^{S},R_{i\,j}^{T}\right)$$

Finally, the multi-representation vector ${\left[R_{i\,j}^{S}\right]}_{j\,1}^{r}$ is connected into a new vector and fed into the classifier module *cls*(⋅) to realize the emotion classification of the EEG signal. Details of these modules are described below.

### Source Domain Selection

Most existing domain adaptation methods simply aggregate all source domain data into one source domain, which often destroys the edge distribution of the source domain, or increases the difficulty of adaptation. Considering the practicality of the model, if the data of a single participant in a single session is simply divided into a source domain, this will greatly increase the complexity of the model and increase the number of parameters. Therefore, based on the above assumptions, we combine two different EEG data from one subject in one session to form a source domain, which not only satisfies the presumption that domain adaptation has a certain source marginal distribution, but also reduces the complexity of the model.

### Common Feature Extractor

This module is used to map the original data of the source domain and the target domain to a common shared latent space, and then extract the low-level features of the two respectively, which are some low-level domain invariant features that represent similar characteristics of different EEG data.

### Multi-Domain Specific Feature Extractor

After obtaining low-level features with domain invariance, we build N multi-domain specific feature extractors corresponding to N source domains. Each multi-domain specific feature extractor has r different domain-invariant feature extractors for learning multiple domain-invariant representations. Since diverse neural network structures can extract different representations from low-level features, we apply a multi-domain specific feature extractor to map each pair of source and target domain data to r latent spaces, and then in each latent space extract the high-level features of the two separately to obtain more domain-specific information. To apply DA and make this r pair of high-level features with different representations close to the corresponding latent space, we choose MMD to estimate the distance between the two domains in the latent space. The final MMD loss of the source domain is the MMD of all r latent spaces. Each source domain has r potential spaces, thus the final MMD loss is the sum of the MMD losses of all source domains and target domains, which can be expressed by Equation 11.


(11) $$M\,M\,D_{f\,i\,n\,a\,l}=\sum_{i=1}^{N}M\,M\,D_{f\,i\,n\,a\,l}^{i}$$

### Classifier

The multiple high-level feature representations obtained from the multi-domain specific feature extractor are connected into a new vector and sent to the classifier for classification. Each source domain corresponds to a softmax classifier. We use cross-entropy to evaluate classification loss, as shown in Equation 12:


(12) $$L_{c\,l\,s}^{i}=-\sum_{a=1}^{M}c_{a}*l\,o\,g\,\left(p_{a}\right)$$

In summary, we first synthesize N source domains into two sets of 2N data from one subject in one session and consider the last collected single-session single-subject EEG data as the target domain, and then all source domains and target domain data are fed into the general feature extractor to extract low-level features. Next, we use a multi-domain specific feature extractor to map the low-level features of the source and target domains to multiple different latent spaces and apply MMD loss to measure the high-level feature representations of the source and target domains in various latent spaces. Finally, we connect different expressions into a new vector and send it to the classifier for classification. The final loss function can be expressed as Equation 13:


(13) $${\text{L}}_{\text{loss}}^{\text{i}}=L_{c\,l\,s}^{i}+\alpha \,M\,M\,D_{f\,i\,a\,n\,l}^{i}$$

The total loss function of all source and target domain data can be defined as Equation 14:


(14) $$L_{l\,o\,s\,s}=\sum_{i=1}^{N}{\text{L}}_{\text{loss}}^{\text{i}}$$

The training is based on Equation 14 and follows the algorithm shown in Algorithm 1. Minimizing this formula is to minimize the classification loss and MMD loss so that the distance between the source domain and the target domain can be as small as possible in different potential spaces, and the classification prediction is as close as possible to the actual label.

**ALGORITHM 1:** Overview of MSMRA module.

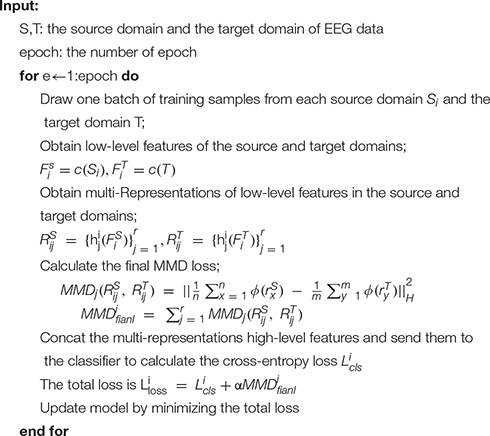

## Experiments

In our experiment, we use the public SEED dataset and SEED-IV dataset to classify emotions. In addition, we not only compare our proposed method with the baseline method but also conduct a large number of ablation experiments and exploratory experiments.

### Implementation Details

**FIGURE 4:**
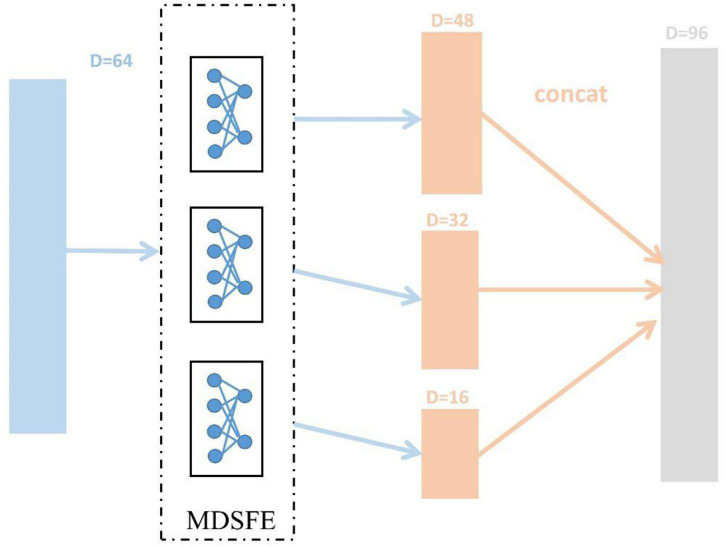
Extract 48-dimensional, 32-dimensional, and 16-dimensional high-level feature representations process from 64-dimensional low-level features, respectively.

The SEED dataset and SEED-IV dataset are first pre-processed (mentioned in section “EEG Data Pre-processing”) to obtain DE features, and reshaped into trials (SEED 15, SEED-IV 24) × 310 × samples. Then we normalize the dimensions of samples, and the ablation experiment in section “Ablation Study” will confirm the effect. After that, we divide the data into N source domains and one target domain in pairs in the manner mentioned in section “Methods.” As described in section “Methods,” the various modules of MSMRA have many details. For the common feature extraction module, since we do not have as many samples as the original data, so in this layer, we take a simple three fully connected layers, which reduce the dimension from 310 (62 × 5) to 64, each fully connected layer is followed by a LeakyReLU activation function (Xu et al., 2015). We also evaluated the ReLU activation function (Nair and Hinton, 2010). Since EEG data is very sensitive, using ReLU will delete many values less than 0, which often loses a lot of information, so we choose LeakyReLU as the activation function. For the multi-domain specific feature extractor module, we use 3 fully connected layers to downsample the low-level feature vector from 64-dimensional to 48-dimensional, 32-dimensional, and 16-dimensional high-level feature representations and then connect them into a vector, as shown in Figure 4. Like the common feature extraction module, 3 fully connected layers are followed by a LeakyReLU activation function. The vector connected by the high-level features is fed into the final classifier, which is a simple fully connected layer, reducing the number of emotion categories (SEED 3, SEED-IV 4) corresponding to the dataset from 96 dimensions. During the network training process, the initial value of the learning rate is 0.01, the batch size is 256, and the epoch is set to 50. The Adam optimizer is used for gradient descent (Kingma and Ba, 2014). In addition, we dynamically adjust the coefficient to achieve the effect of giving priority to the classification results, and the coefficient can be expressed as Equation 15.


(15) $$\alpha =\frac{2}{1+{\text{e}}^{-10^{*}\,i/\mathrm{epoch}}}-1$$

### BaseLines

We compare the MSMRA model with the latest various competitors on the SEED dataset and SEED-IV dataset, including Deep domain confusion (DDC) (Tzeng et al., 2014), which treats the MMD algorithm as an adaptive metric, domain adaptation model (DAN) (Li et al., 2018), which adopts deep adaptation network to EEG-based emotion recognition, Deep coral (DCORAL) (Sun and Saenko, 2016), which aligns the second-order statistical features of the source domain and target domain distribution by linear transformation method, Domain-Adversarial Training of Neural Networks (DANN) (Ganin et al., 2017), which introduces the idea of adversarial network to the field of transfer learning, Plug-and-play domain adaptation (PPDA) (Zhao et al., 2021), which proposes a plug-and-play domain adaptation method for reducing the calibration time. Dynamical Graph Convolutional Neural Networks (DGCNN) (Song et al., 2018) and Multisource Marginal Distribution Adaptation (MS-MDA) (Chen et al., 2021) which applies a multi-source domain method.

### Results

All the experimental results of the two data sets are shown in Tables 3, 4, respectively. In the experiment, except for some results directly taken from the original paper (MS-MDA, DGCNN), all the hyperparameters are the same. From these results, we have the following profound observations.

• In the SEED data set, our proposed method MSMRA surpasses most of the current state-of-the-art methods in Cross-subject and Cross-session scenarios. It can be seen that our method improves the accuracy of at least close to 2% compared to other methods in the Cross-session scene, and for the first time exceeded the 90% accuracy rate, as far as we know. In the Cross-subject scenario, although our method is 2% lower than MS-MDA, it is better than all other methods, which shows that our method is competitive.
• On the SEED-IV dataset, it can be clearly seen that our proposed method significantly exceeds all other competitors, and each has improved by at least 11 and 10% compared to others in Cross-session and Cross-subject scenarios, it is a piece of exciting news. From the results, we can infer that our method can also achieve good classification results on small sample datasets because multiple high-level features containing rich information are extracted from the MDSFE module.

**TABLE 3:** Comparisons of the average accuracies and standard deviations of cross-session and cross-subject scenarios on SEED database among the various methods.

Dataset	Method	Cross-session	Cross-subject
	DGCNN	–	79.95 ± 9.02
	DDC	81.53 ± 6.83	68.99 ± 3.23
	DAN	79.93 ± 7.06	65.84 ± 2.25
SEED	DCORAL	76.86 ± 7.61	66.29 ± 4.53
	DANN	–	79.19 ± 13.14
	PPDA	–	86.70 ± 7.10
	MS-MDA	88.56 ± 7.80	**89.63** ± **6.79**
	MSMRA (Ours)	**90.30** ± **5.26**	87.62 ± 7.53
			

*Bold indicates the maximum average accuracies of cross-session and cross-subject scenarios among the various methods.*

**TABLE 4:** Comparisons of the average accuracies and standard deviations of cross-session and cross-subject scenarios on SEED-IV database among the various methods.

Dataset	Method	Cross-session	Cross-subject
	DDC	57.63 ± 11.28	37.71 ± 6.36
	DAN	55.14 ± 12.79	32.44 ± 9.02
SEED-IV	DCORAL	44.63 ± 11.38	37.43 ± 3.08
	MS-MDA	61.43 ± 15.71	59.34 ± 5.48
	MSMRA (Ours)	**72.38** ± **10.12**	**69.77** ± **7.37**

*Bold indicates the maximum average accuracies of cross-session and cross-subject scenarios among the various methods.*

### Ablation Study

To further understand the MSMRA model we proposed, we performed the ablation experiments of removing the initial normalization of the data and removing the MDSFE module on the SEED and SEED-IV datasets respectively, and evaluate the performance of the ablated model. The hyperparameters of all ablation experiments are consistent, and the experimental results are shown in Table 5. As shown in the table, the first row of the two datasets is the complete method we proposed, and we can see that the accuracy rate is the highest; the second row removes the initial data normalization, which makes the model prone to be affected by outliers resulting in a decrease in accuracy; the third row removes the multi-domain specific feature extraction module, which weakens the feature representation ability of the model. In the fourth row, the initial data normalization module and the multi-domain specific feature extraction module are removed. It can be seen that the experimental accuracy is lower than the removal of a single ablation module. Moreover, notice that although a certain module is removed, our proposed method is still competitive with other methods.

**TABLE 5:** Ablation study of MSMRA on SEED and SEED-IV.

Dataset	Method	Cross-session	Cross-subject
SEED	Ours full	**90.30** ± **5.26**	**87.62** ± **7.53**
	w/o normalization	80.21 ± 9.95	84.12 ± 6.17
	w/o MDSFE	89.55 ± 5.17	87.17 ± 5.41
	w/o normalization + MDSFE	77.02 ± 11.11	80.88 ± 7.22
SEED-IV	Ours full	**72.38** ± **10.12**	**69.77** ± **7.37**
	w/o normalization	43.39 ± 6.97	52.27 ± 5.18
	w/o MDSFE	71.97 ± 12.53	60.19 ± 9.60
	w/o normalization + MDSFE	43.33 ± 4.79	50.94 ± 3.24

*Bold indicates the maximum average accuracies of cross-session and cross-subject scenarios among the various methods.*

## Conclusion

In general, the current emotion classification based on EEG signals is hindered due to the instability of the internal data of Cross-session and Cross-subject when collecting data. Domain adaptation increases the classification accuracy of cross-domain data by finding a mapping relationship to map the source domain and target domain to a latent space, and minimizing the distance between the two. Based on the above, we propose a multi-source and multi-representation domain adaptive network (MSMRA), which regards EEG signals as multiple source domains rather than simply as one source domain, which satisfies the assumption that the source domain data has a certain marginal distribution. It can be seen from Tables 3, 4 that the classification accuracy of our proposed method significantly exceeds that of the latest single-source domain method. Since the multi-source domain method is easy to cause the network to be bloated and complicated, we propose a pairwise combination method to solve this problem. Moreover, considering that the representation distribution extracted in a single structure of aligning may only contain partial information, we propose a multi-domain specific feature extraction module, which can extract multiple high-level features of different dimensions. Table 5 shows that this module is helpful to improve the accuracy of emotion classification. Future work will focus on how to divide multi-source domains and how to extract more effective multi-domain specific features.

## Data Availability Statement

Publicly available datasets were analyzed in this study. This data can be found here: https://bcmi.sjtu.edu.cn/~seed/index.html.

## Ethics Statement

Ethical review and approval was not required for the study on human participants in accordance with the local legislation and institutional requirements. The patients/participants provided their written informed consent to participate in this study. Written informed consent was obtained from the individual(s) for the publication of any potentially identifiable images or data included in this article.

## Author Contributions

JC wrote the main manuscript and conducted the experiments. XH and CY participated in the writing of the manuscript and modified the English grammar of the article. SC and ZL made the experiments. ZW analyzed the results. All authors reviewed the manuscript.

## Conflict of Interest

The authors declare that the research was conducted in the absence of any commercial or financial relationships that could be construed as a potential conflict of interest.

## Publisher’s Note

All claims expressed in this article are solely those of the authors and do not necessarily represent those of their affiliated organizations, or those of the publisher, the editors and the reviewers. Any product that may be evaluated in this article, or claim that may be made by its manufacturer, is not guaranteed or endorsed by the publisher.
